# Novel nutraceutical supplements with yeast β-glucan, prebiotics, minerals, and *Silybum marianum* (silymarin) ameliorate obesity-related metabolic and clinical parameters: A double-blind randomized trial

**DOI:** 10.3389/fendo.2022.1089938

**Published:** 2023-01-27

**Authors:** Victor Nehmi-Filho, Aline Boveto Santamarina, Jéssica Alves de Freitas, Ericka Barbosa Trarbach, Daniela Rodrigues de Oliveira, Fanny Palace-Berl, Erica de Souza, Danielle Araujo de Miranda, Antonio Escamilla-Garcia, José Pinhata Otoch, Ana Flávia Marçal Pessoa

**Affiliations:** ^1^ Natural Products and Derivatives Laboratory (LIM-26), Department of Surgery, University of São Paulo Medical School, São Paulo, SP, Brazil; ^2^ Research and Development Efeom Nutrition S/A, São Paulo, SP, Brazil; ^3^ Department of Biosciences, Federal University of São Paulo (UNIFESP), Santos, SP, Brazil; ^4^ Laboratory of Cellular and Molecular Endocrinology (LIM25), Division of Endocrinology and Metabology, Clinics Hospital, University of São Paulo Medical School, São Paulo, SP, Brazil; ^5^ Department of Pharmacology & Toxicology, Medical College of Wisconsin, Milwaukee, WI, United States; ^6^ Monte Azul Ambulatory, São Paulo, SP, Brazil; ^7^ Departament of Physiology, Escola Paulista de Medicina/Universidade Federal de São Paulo, São Paulo, SP, Brazil; ^8^ University Hospital of the University of São Paulo, University of São Paulo Medical School, São Paulo, SP, Brazil; ^9^ Natural Products Committee, Brazilian Academic Consortium for Integrative Health (CABSIN), São Paulo, Brazil

**Keywords:** nutraceutics, supplement, *Silybum marianum*, prebiotic, obesity, endocrine parameters, transaminases

## Abstract

**Purpose:**

It is known that obesity has a multifactorial etiology that involves genetic and environmental factors. The WHO estimates the worldwide prevalence of 1.9 billion overweight adults and more than 650 million people with obesity. These alarming data highlight the high and growing prevalence of obesity and represent a risk factor for the development and aggravation of other chronic diseases, such as nonalcoholic fatty liver disease (NAFLD) that is frequently considered the hepatic outcome of type 2 diabetes. The use of non-pharmacological therapies such as food supplements, nutraceuticals, and natural integrative therapies has grown as an alternative tool for obesity-related diseases compared to conventional medications. However, it is a still little explored research field and lacks scientific evidence of therapeutic effectiveness. Considering this, the aim is to evaluate whether a new nutraceutical supplement composition can improve and supply essential mineral nutrients, providing an improvement of obesity-related metabolic and endocrine parameters.

**Methods:**

Sedentary volunteers (women and men) with body mass index (BMI) ≤34.9 kg/m^2^ were divided into two groups: Novel Nutraceutical Supplement_(S) (n = 30) and Novel Nutraceutical Supplement (n = 29), differing in the absence (S) or presence of silymarin, respectively. Volunteers were instructed to take two capsules in the morning and two capsules in the evening. No nutritional intervention was performed during the study period. The data (anthropometrics and anamneses) and harvest blood (biochemistry and hormonal exams) were collected at three different time points: baseline time [day 0 (T0)], day 90 (T90), and day 180 (T180) post-supplementation.

**Results:**

In the anthropometric analysis, the waist circumference in middle abdomen (WC-mid) and waist circumference in iliac crest (WC-IC) were reduced. Also, the waist-to-height ratio (WHt R) and waist-to-hip ratio (WHR) seem to slightly decrease alongside the supplementation period with both nutraceutical supplements tested as well as transaminase enzyme ratio [aspartate aminotransferase (AST)/alanine aminotransferase (ALT) ratio (AAR)], a known as a biomarker of NAFLD, and endocrine hormones cortisol and thyroid-stimulating hormone (TSH) at 90 and 180 days post-supplementation.

**Conclusions:**

In a condition associated with sedentary and no nutritional intervention, the new nutraceutical supplement composition demonstrated the ability to be a strong and newfangled tool to improve important biomarkers associated with obesity and its comorbidities.

## 1 Introduction

The etiology of chronic non-communicable diseases such as obesity is multifactorial, including genetics and the environment (1). These diseases are the world’s leading cause of mortality (2, 3). Data from the World Health Organization (WHO) estimate the worldwide prevalence of 1.9 billion overweight adults [body mass index (BMI) ≥25 kg/m²] and more than 650 million people with obesity (BMI ≥30 kg/m²) (3). Recently, during the critical phase of the pandemic, the prevalence of chronic non-communicable diseases was closely related to higher mortality in hospitalization due to coronavirus disease 2019 (COVID-19) (4). These alarming data highlight the high and growing prevalence of obesity that represents a risk factor for the development and aggravation of other metabolic diseases.

Considering this, the incessant search for nutritional intervention strategies is essential to pursue the mitigation of obesity and its comorbidities. In this sense, the use of natural products and derivatives as non-pharmacological strategies can be an alternative in the prevention and treatment of acute or chronic diseases. These tools could contribute to preventing or reducing obesity-related negative outcomes, promoting longer life spans and better-quality lives (5). Thus, the use of food supplements, nutraceuticals, and natural integrative therapies has grown compared to conventional medications, which are still necessary and indispensable for the adequate treatment of some conditions (6, 7). Indeed, non-pharmacological therapies seem to be promising strategies for the prevention and treatment of metabolic–inflammatory diseases, such as obesity, metabolic syndrome, dyslipidemia, and type 2 diabetes (8–10).

Nevertheless, in this study, we also intend to contribute to the construction of knowledge for clinical practice using anthropometric assessment and serum tests reachable to practitioners’ daily routines. Previous studies have shown that increased neck circumference in obese adults is related to increased cardiovascular and inflammatory risk, acting as an anthropometric predictor of cardiovascular and inflammatory risk (11). Also, the waist-to-height ratio (WHtR), a measure with significant statistical representation, works as a good predictor of metabolic syndrome and appears to be directly proportional to inflammatory levels and correlated with poor eating habits (12–14).

Several cellular mechanisms are involved in obesity pathologies such as the metabolic pathways activated by directly influencing the insulin signaling in insulin-target tissues such as the liver, eventually leading to insulin resistance (15). Thus, nonalcoholic fatty liver disease (NAFLD) is frequently considered the hepatic outcome of type 2 diabetes. Indeed, insulin resistance also plays a central role in the development of the fatty liver (16), since hepatic fatty acid oxidation is reduced by mitochondrial dysfunctions triggered by obesity, resulting in hepatic accumulation of triacylglycerols (17, 18). Another common metabolic alteration in obesity is persistent and systemic low-grade inflammation (19, 20), which has been termed metainflammation. Metainflammation is closely linked to insulin resistance mechanisms that feedback each other, perpetuating long-term metabolic dysfunction (21). The combination of low-grade inflammation processes and insulin resistance establishment is an important link between obesity and its comorbidities.

The increased incidence and prevalence of obesity-related comorbidities is a concern still unsolved by worldwide public health regulators. Despite efforts to mitigate obesity, little effect has been noticed on the increase of all diseases associated with it (22). Considering this, the benefits of several natural compounds have been investigated in the obesity research field nowadays. In this sense, the effect of isolated prebiotic compounds, such as fructooligosaccharides (FOSs) (23), galactooligosaccharides (GOSs) (24), and yeast β-glucans (25, 26), has been related to improved metabolic markers recovering the health status. Also, supplementation with minerals such as magnesium (27), zinc (28), and selenium (29) and plant-derived compounds such as *Silybum marianum* (silymarin) (30) has displayed positive effects in obesity models. Compounds such as FOS and GOS display a potential effect of modulating the gut microbiome, which is closely linked to inflammatory modulation (31). The same aspect might be related to yeast β-glucans (32), magnesium (27), zinc (28), and selenium (33), which are known for the immune system-strengthening action. Likewise, silymarin, the bioactive compound obtained from milk thistle (*S. marianum*), has been used over the centuries to primarily treat liver diseases and protect the liver against obesity injury, contributing to the restoration of hepatic function (34). The main bioactive compound of silymarin is the component called silybin. Silybin is a flavonolignan that has different activities such as antioxidant and anti-inflammatory properties (35). In this sense, preclinical protocols testing a combination of these compounds have shown their benefits in the improvement of mitochondrial activity and key inflammatory molecules involved in the pathogenesis of several non-communicable chronic diseases such as type 2 diabetes, obesity, and cardiovascular diseases (9, 10). The preclinical model compared the effect of the supplement components separately and in combination, and findings suggest that the nutraceutical compounds might have a stronger synergic effect, representing an innovative tool for obesity and insulin resistance management.

Therefore, in this study, we aim to apply our previously developed supplement composition containing yeast β-glucan, prebiotics, minerals, and *S. marianum* (milk thistle or silymarin) (9, 10) as a tool to modulate inflammatory and metabolic responses in a population with obesity. The main hypothesis is that the supplements can improve and supply essential micronutrients such as zinc, magnesium, and selenium, which are usually deficient in populations with obesity (36), providing an improvement of fatty liver diseases through metabolic and endocrine pathways.

## 2 Materials and methods

### 2.1 Ethics committee approval

This work was approved by the HC-FMUSP Research Ethics Committee under the CAAE number 39984320.5.0000.0068. This research project was carried out following the relevant guidelines and regulations and was approved by the Ethics Committee for the Analysis of Research Projects (CAPPesq). This study received approval from the Brazilian National System of Genetic Registration (SisGen), under the number AC29D69, and registered as a clinical trial under identification number NCT04810572 (ClinicalTrials.gov).

### 2.2 Recruitment of volunteers and experimental design

The “Novel Nutraceutical Supplement Trial” is a randomized controlled trial addressing sedentary people with three assessment points (baseline and 90 and 180 days post-supplementation) from Monte Azul Ambulatory, São Paulo, SP, Brazil, and online invitation between March and June 2021 (n = 133). Stratified randomization was applied to ensure a balance of baseline key factors between groups. In this study, age, BMI, and gender were considered baseline characteristics for sample stratification before randomization. The pharmaceutical team responsible for preparing the supplement capsules was the blind spot between participants and researchers until the end of the study. The sample size enrolled was based on a calculation determined using the G*Power software (37). Assuming an F test with a type I error of 5% at 95% power and a success rate (effect size) of 0.2, the required total sample size was 66. Due to the long-term period of supplementation protocol and the progress of the COVID-19 pandemic during the protocol, to account for a potential dropout of 20%, we inflate this to at least 80 participants.

The inclusion criteria were as follows: healthy volunteers of both genders aged between 44 and 64 years; BMI ≤34.9 kg/m^2^; no diet intervention; and sedentary lifestyle. To guarantee that the volunteers had a sedentary lifestyle, we applied International Physical Activity Questionnaire (IPAQ)-Short Version (38) and presented it in **Table 1**. The exclusion criteria were as follows: volunteers who are allergic to some of the components of the nutraceutical formulation; the use of insulin, corticoids, and non-steroidal anti-inflammatory drugs for more than 15 days; AIDS; hepatitis; pregnancy; and patients under treatment with chemotherapy.

**Table 1:** Population physical activity level is classified by the International Physical Activity Questionnaire (IPAQ).

Physical activity level	Novel Nutraceutical Supplement_(S)			Novel Nutraceutical Supplement		
	T0	T90	T180	T0	T90	T180
**Inactive**	63.33% (n=19)	63.33% (n=19)	66.66% (n=20)	62.06% (n=18)	62.06% (n=18)	62.06% (n=18)
**Minimally active**	36.66% (n=11)	36.66% (n=11)	33.33% (n=10)	37.93% (n=11)	37.93% (n=11)	37.93% (n=11)
**HEPA active**	-	-	-	-	-	-

HEPA active, health-enhancing physical activity.

All volunteers signed a term of free and informed consent before starting the study and could withdraw the consent at any time. Therefore, volunteers were divided into two groups: Novel Nutraceutical Supplement_(S) (n = 30) and Novel Nutraceutical Supplement (n = 29). Volunteers were instructed to take two capsules of the supplements in the morning and two capsules in the evening. The participants were instructed to maintain their eating habits, and no nutritional intervention was performed during the study period.

**Figure 1:**
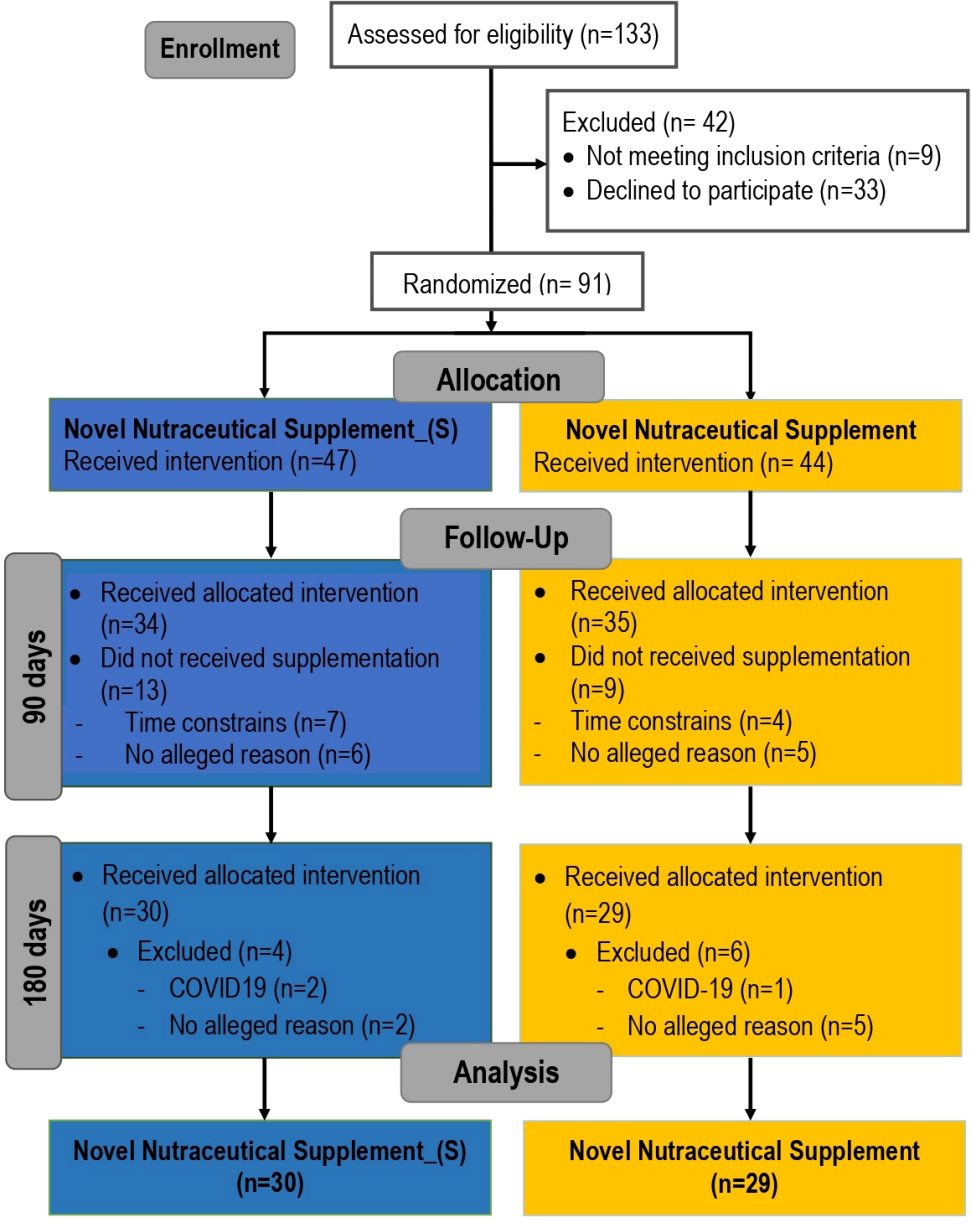
Consolidated Standards of Reporting Trials (CONSORT) flowchart describing the recruitment of volunteers and the experimental design carried out on this clinical trial.

We used a Consolidated Standards of Reporting Trials (CONSORT) (39) flow diagram to display the trial. Initially, during the enrollment process, a total of 133 volunteers were assessed. At that stage, 42 people were excluded because they did not meet the inclusion criteria (n = 9) or declined to participate (n = 33). Thus, 91 participants were randomized into the two experimental groups, the Novel Nutraceutical Supplement_(S) group (n = 47) and the Novel Nutraceutical Supplement group (n = 44), and initiated the supplementation period. During the study follow-up at the T90 in the Novel Nutraceutical Supplement_(S) group, 13 participants declined the protocol because of time constraints (n = 7) or for no alleged reason (n = 6) and 34 participants kept on with the study. In the Novel Nutraceutical Supplement group at T90, a total of nine participants declined the study because of time constraints (n = 4) or for no alleged reason (n = 5) and 35 participants were followed in the study. At T180 on Novel Nutraceutical Supplement_(S), four people were excluded from the protocol for COVID-19 diagnosis (n = 2) or no alleged reasons (n = 2); a total of 30 participants then completed the study. In the Novel Nutraceutical Supplement group, six participants were excluded because of COVID-19 diagnosis (n = 1) or no alleged reasons (n = 5); 29 participants completed the supplementation protocol as demonstrated in the CONSORT flowchart (**Figure 1**). The data and harvested blood were obtained from the volunteers at three different time points: baseline time [day 0 (T0)] and day 90 (T90) and day 180 (T180) post-supplementation as shown in **Table 2**.

**Table 2:** Anthropometric and demographic characterization of the study population.

	Novel Nutraceutical Supplement_(S)			Novel Nutraceutical Supplement			*p*
**Variables**	**T0**	**T90**	**T180**	**T0**	**T90**	**T180**
**Sample Size**	30			29			
**Age (years)**	54.10 ± 5.52			54.83 ± 4.59			–
**Height (cm)**	162.0 (153.0-171.0)			161.5 (155.8-186.0)			–
**Body Mass (kg)**	72.10 ± 12.50	72.17 ± 11.69	72.44 ± 12.34	73.47 ± 12.34	73.85 ± 12.47	74.03 ± 12.64	–
**BMI (kg/m^2^)**	27.23 ± 3.28	27.29 ± 3.10	27.38 ± 3.31	27.65 ± 3.45	27.80 ± 3.58	27.88 ± 3.64	–
**Neck (cm)**	35.55 ± 3.16	35.70 ± 4.06	35.03 ± 3.08	36.12 ± 3.02	35.94 ± 3.51	35.78 ± 3.34	–
**WC-mid (cm)**	90.68 ± 9.64	89.77 ± 9.76	88.89 ± 9.82	92.12 ± 10.66	91.64 ± 11.00	90.53 ± 10.48	0.010^a^; 0.030^b^
**Hip (cm)**	105.29 ± 6.88	103.74 ± 8.52	104.79 ± 7.00	104.54 ± 7.01	104.15 ± 8.06	105.00 ± 7.21	–
**WC-IC (cm)**	99.46 ± 8.25	100.18 ± 8.14	99.74 ± 9.25	100.06 ± 8.44	102.76 ± 8.20	99.81 ± 9.01	0.010^c^; <0.010^d^
**WHtR**	0.57 ± 0.59	0.56 ± 0.06	0.55 ± 0.05	0.59 ± 0.06	0.57 ± 0.06	0.56 ± 0.06	–
**WHR**	0.86 ± 0.07	0.87 ± 0.08	0.87 ± 0.07	0.91 ± 0.07	0.88 ± 0.07	0.88 ± 0.09	–

BMI, body mass index; WC-mid, waist circumference in middle abdomen; WC-IC, waist circumference in iliac crest; WHtR, waist-to-height ratio; WHR, waist-to-hip ratio.

Data values are expressed as mean ± SEM.

**^a^** Significant difference Supplement_(S) T0 vs. T180.

**^b^** Significant difference Supplement_(S) T90 vs. T180.

**^c^** Significant difference Novel Supplement T0 vs. T90.

**^d^** Significant difference Novel Supplement T90 vs. T180.

T0, day 0; T90, 90 days post-supplementation; T180, 180 days post-supplementation.

### 2.3 Novel nutraceutical supplement formulations

We prepared two formulations (Patent number: BR 10 2020 016156 3): Formulation 1 (n = 30): Novel Nutraceutical Supplement_(S) (without silymarin), developed and tested in the present study, contained the following components: zinc (Zn) 1%, magnesium (Mg) 1% (Purifarma Distribuidora Quımica e Farmacêutica, São Paulo, Brazil), FOSs 45% (NutraFlora®, Westchester, Illinois, USA), selenomethionine (Se) 0.01%, GOSs 10%, tixosil 5%, and 1.3/1.6-(b-glycosidic bonds) yeast b-glucans (Saccharomyces cerevisiae) 6% (Biorigin, São Paulo, Brazil); and Formulation 2 (n = 29): zinc (Zn) 1%, magnesium (Mg) 1% (Purifarma Distribuidora Química e Farmacêutica, São Paulo, Brazil), FOSs 45% (NutraFlora®, Westchester, Illinois, USA), selenomethionine (Se) 0.01%, GOSs 10%, tixosil 5%, 1.3/1.6-(b-glycosidic bonds) yeast b-glucans (Saccharomyces cerevisiae) 6% (Biorigin, São Paulo, Brazil), and S. marianum (silymarin extract 9%) (SM Empreendimento Farmacê utica LTDA, São Paulo, Brazil). The percentages of the Novel Nutraceutical Supplement were determined following recommendations and parameters published by the European Food Safety Authority (EFSA) (40). The formulations of the novel nutraceutical supplements were manipulated by the compounding pharmacy “Solis Magistral Farmácia Homeopática Sensitiva (São Paulo, Brazil),” which kept the double blinding point of this clinical study.

### 2.4 Anthropometric parameters

Anthropometric parameters were collected and analyzed at baseline time (T0) and T90 and T180 post-supplementation. The volunteers were weighed using the Body Composition Scale 2 (Xiaomi Mi, Beijing, China). The hip (cm), iliac crest (cm), waist (cm), and neck (cm) circumferences and height (cm) were measured using a plastic tape measurer. BMI was calculated by dividing weight (kg) by the square of height (m).

### 2.5 Aspartate aminotransferase (AST) and alanine aminotransferase (ALT) ratio (AAR), biochemistry, and endocrine parameters

The volunteers’ blood sample was collected on T0 and T90 and T180 post-supplementation. All samples were collected between 7:00 a.m. and 9:00 a.m. in the morning. The samples were processed on the same day of collection to immediately perform biochemical analysis and endocrine parameters. Total cholesterol, triglycerides (TGs), non-high-density lipoprotein (HDL)-cholesterol, low-density lipoprotein (LDL)-cholesterol, very-low-density lipoprotein (VLDL)-cholesterol, HDL-cholesterol, aspartate aminotransferase (AST), alanine aminotransferase (ALT), alkaline phosphatase, gamma-glutamyl transferase (gamma-GT), creatinine, insulin, cortisol, thyroxine (T4), thyroid-stimulating hormone (TSH), and C-reactive protein (CRP) were analyzed in the serum, and plasma glucose levels obtained. Biochemical analyses and endocrine parameters were measured at the partner laboratory “Fleury Medicina e Saúde.” AST/ALT ratio (AAR) was calculated according to Rief et al. (41). Atherogenic index (AI) was determined following the formula AI = [log10(TAG÷HDL-c)].

### 2.6 Statistical analysis

All statistical analyses were conducted following the CONSORT recommendations (39). Data were classified as parametric or nonparametric based on the Shapiro–Wilks test. Parametric data are represented as mean ± standard deviation, and nonparametric data are represented as median and interquartile range. Comparisons between groups involving parametric data were performed with repeated-measures analysis of variance (ANOVA). Comparisons between groups involving nonparametric data were performed using the Friedman test. Analyses were performed using STATA^®^ 14.0 (Stata Corp. LCC, College Station, TX, USA) and GraphPad Prism 9.0 (GraphPad Software, La Jolla, CA, USA) software.

## 3 Results

### 3.1 Novel nutraceutical supplements reduce anthropometric parameters

Accessing the data of the participant’s physical activity level evaluated by the IPAQ-Short Version, we can notice that the sample is composed mostly of inactive people, with a small percentage of minimally active people and without individuals practicing health-enhancing physical activity (HEPA active). This characteristic was maintained for both groups throughout the study period, as shown in **Table 1**. Analyzing the population characterization is noteworthy for the homogeneity between groups through age, height, body mass, and BMI, displaying similar data. Regarding the waist circumference, in the middle abdomen (WC-mid), we have found a significant reduction in the Supplement_(S) group at T0 and T90 compared to T180. We also have found an increase in waist circumference in the iliac crest (WC-IC) at T0 vs. T90 followed by a significant reduction at T90 compared to T180 in the novel supplement group. The BMI, neck, and hip circumference did not differ among the evaluations. Despite the absence of a statistical difference, the WHtR and waist-to-hip ratio (WHR) seem to slightly decrease alongside the supplementation period in both groups. These data are presented in **Table 2**.

### 3.2 Lipid profile under the nutraceutical supplement effect

**Figure 2:**
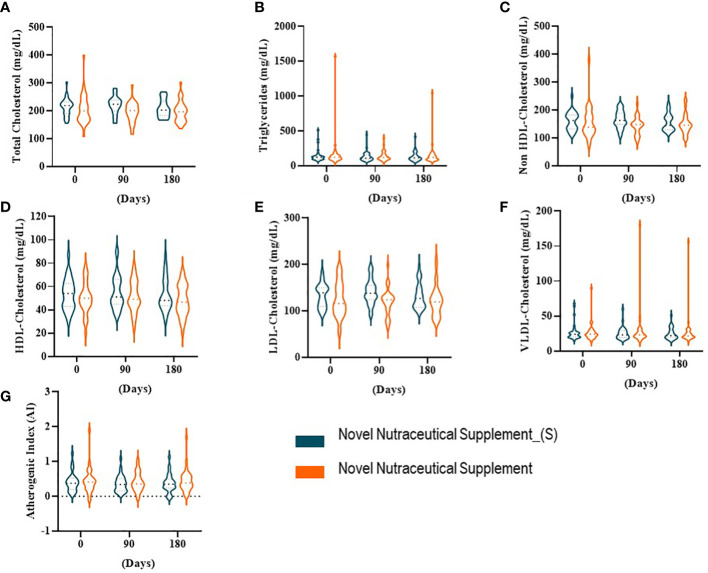
Lipid profile at baseline time (Day 0) and 90 and 180 days post-supplementation. **(A)** Total cholesterol. **(B)** Triglycerides. **(C)** Non-HDL-cholesterol. **(D)** HDL-cholesterol. (E) LDL-cholesterol. **(F)** VLDL-cholesterol. **(G)** Atherogenic index (AI). Novel Nutraceutical Supplement_(S) (n = 30) and Novel Nutraceutical Supplement (n = 29). Values are expressed as mean ± SEM or median (min to max, box plot) by plot violin graphic. HDL, high-density lipoprotein; LDL, low-density lipoprotein; VLDL, very-low-density lipoprotein.

We evaluated the serum total cholesterol, TGs, and lipoproteins in all volunteers who received the Novel Nutraceutical Supplement_(S) (n = 30) and the Novel Nutraceutical Supplement (n = 29) (**Figure 1**). Neither of the supplements changed the total cholesterol (**Figure 2A**), TGs (**Figure 2B**), non-HDL-cholesterol (**Figure 2C**), HDL-cholesterol (**Figure 2D**), LDL-cholesterol (**Figure 2E**), VLDL-cholesterol (**Figure 2F**), and AI (**Figure 2G**) at 90 and 180 days post-supplementation.

### 3.3 Novel nutraceutical supplements modulate biomarkers associated with non-alcoholic fatty liver disease (NAFLD)

**Figure 3:**
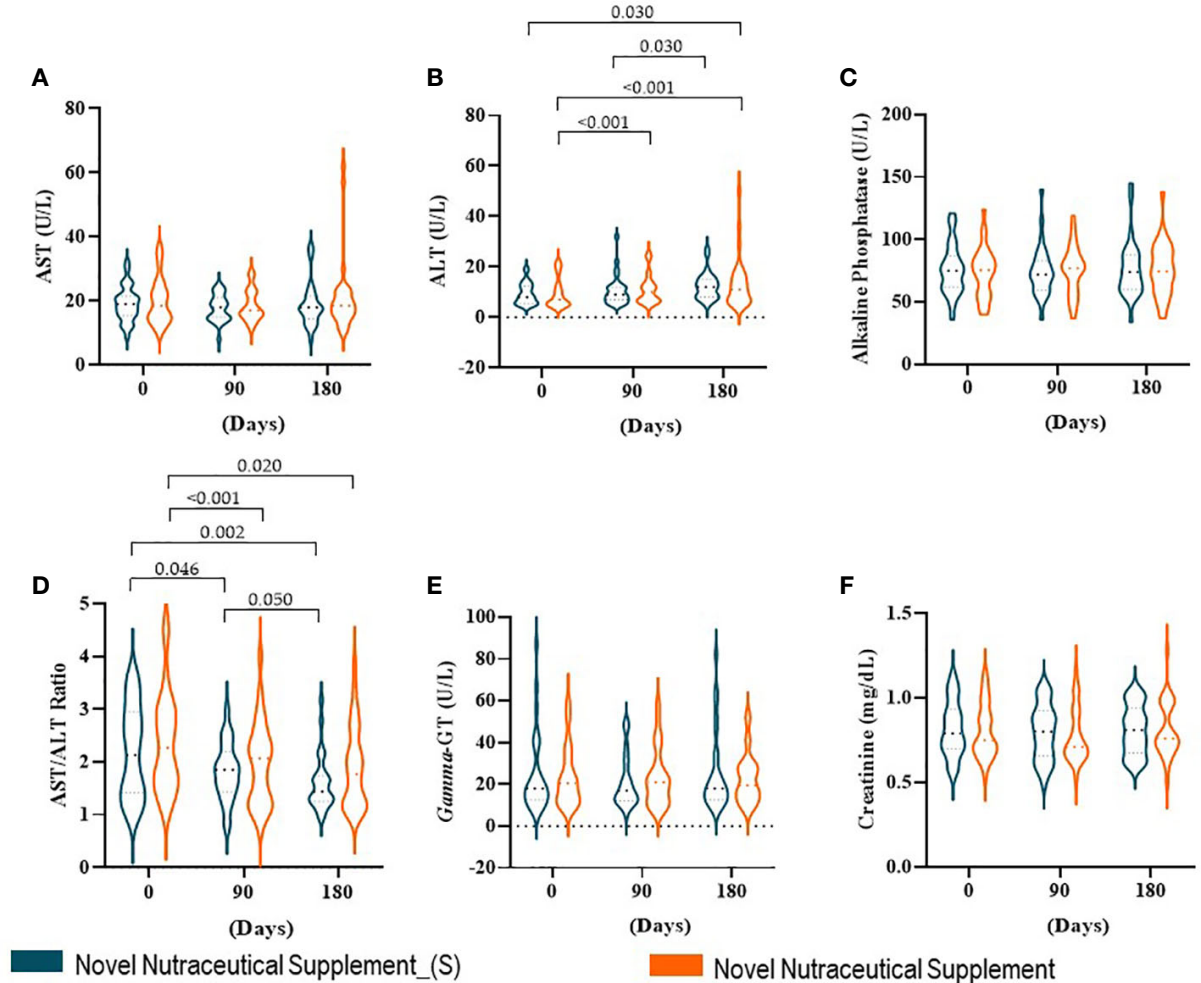
Liver enzymes, AST/ALT ratio (AAR), and creatinine at baseline time (Day 0) and 90 and 180 days post-supplementation. **(A)** AST. **(B)** ALT. **(C)** Alkaline phosphatase. **(D)** AAR. **(E)** Gamma-GT. **(F)** Creatinine. Novel Nutraceutical Supplement_(S) (n = 30) and Novel Nutraceutical Supplement (n = 29). Values are expressed as mean ± SEM or median (min to max, box plot) by plot violin graphic. AST, aspartate aminotransferase; ALT, alanine aminotransferase; gamma-GT, gamma-glutamyl transferase.

In our cohort, liver and renal functions were estimated by liver enzymes and creatinine, respectively. The liver enzymes AST (**Figure 3A**), alkaline phosphatase (**Figure 3C**), and gamma-GT (**Figure 3E**), even creatinine (**Figure 3F**), did not show differences between baseline time and 90 and 180 days post-supplementation in both groups. However, the ALT enzyme (**Figure 3B**) in Nutraceutical Supplement_(S) increased only 180 days post-supplementation as compared with 90 days post-supplementation. While in the Novel Nutraceutical Supplement group, we observed that the ALT enzyme (**Figure 3B**) increased at 90 and 180 days post-supplementation as compared with baseline time. An elevated AAR (**Figure 3D**) (above 1.0) has been associated with fibrosis/cirrhosis prevalence in NAFLD (41). All groups in baseline time demonstrate elevated AAR (>2.20) (**Figure 3D**), and Novel Nutraceutical Supplement and Supplement_(S) were able to decrease AAR at 90 and 180 days post-supplementation.

### 3.4 Carbohydrate metabolism and novel nutraceutical supplements

**Figure 4:**
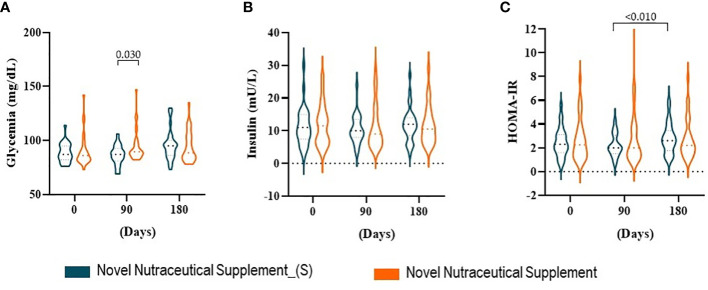
Carbohydrate profile at baseline time (Day 0) and 90 and 180 days post-supplementation. **(A)** Glycemia. **(B)** Insulin. **(C)** HOMA-IR. Novel Nutraceutical Supplement_(S) (n = 30) and Novel Nutraceutical Supplement (n = 29). Values are expressed as mean ± SEM or median (min to max, box plot) by plot violin graphic. HOMA-IR, Homeostatic Model Assessment of Insulin Resistance.

The influence of the supplements on carbohydrate metabolism was measured by glycemia, insulin, and Homeostatic Model Assessment of Insulin Resistance (HOMA-IR). When we analyzed the effects of the supplements on glycemia (**Figure 4A**) and insulin (**Figure 4B**) after 90 and 180 days compared to baseline time (day 0), we did not observe changes. While the Nutraceutical Supplement increased glycemia as compared with Novel Nutraceutical Supplement_(S) at 90 days post-supplementation. HOMA-IR (**Figure 4C**) follows the same pattern compared to the baseline time with the supplementations. Although HOMA-IR increased at the 180-day compared to the 90-day post-supplementation in the Novel Nutraceutical Supplement_(S) group there was no statiscal differences.

### 3.5 Novel nutraceutical supplements influence endocrine hormones

**Figure 5:**
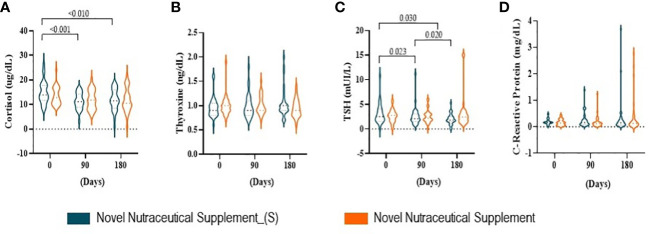
Hormones produced by the adrenal and thyroid gland at baseline time (Day 0) and 90 and 180 days post-supplementation. Novel Nutraceutical Supplement_(S) (n = 30) and Novel Nutraceutical Supplement (n = 29). **(A)** Cortisol. **(B)** Thyroxine (T4). **(C)** TSH. **(D)** C-reactive protein. Values are expressed as mean ± SEM or median (min to max, box plot) by plot violin graphic. TSH, thyroid-stimulating hormone.

Endocrine hormones cortisol, thyroxine (T4), thyroid-stimulating hormone (TSH), and inflammatory marker C-reactive protein were evaluated at 90 and 180 days post-supplementation. Serum cortisol (**Figure 5A**) decreased at 90 and 180 days post the Nutraceutical Supplement_(S) vs. the baseline time (day 0), while the Nutraceutical Supplement was not able to modulate this steroid hormone produced by adrenal glands. The thyroid hormone gland T4 and C-reactive protein (**Figures 5B, D** **)** were not influenced by either of the supplements during the 180 days of supplementation. While TSH (**Figure 5C**) decreased in the Novel Nutraceutical Supplement_(S) group when compared to baseline time at all points evaluated, but not in the Novel Nutraceutical Supplement group.

## 4 Discussion

The application of natural products or their derivatives for the prevention or treatment of several pathologies represents a reasonable and accessible way for clinical practitioners and the general population to use natural resources as viable alternative therapies for health promotion and disease intervention. The development of new products containing different molecules and natural derivatives that interact synergistically has shown beneficial potential, acting holistically and producing few adverse effects on the human body (5). Our recently published findings show the synergistic effects between yeast β-glucan, prebiotic, minerals (selenium, zinc, magnesium), and *S. marianum* of an innovative supplement with nutraceutical properties in a preclinical model of diet-induced obesity (9, 10). These preclinical investigations have shown that after 4 weeks of supplementation, the new nutraceutical reduced fasting glycemia, insulin, HOMA-IR, HOMA-β, dyslipidemia, ectopic fat deposition, and hepatic fibrosis levels. It was probably mediated by molecules such as Peroxisome proliferator- activated receptor gamma (PPARg) coactivator 1 α (PGC-1α), interleukin-6 (IL-6), and IL-10, since our previous preclinical study has shown the modulation of this pathway by gene and protein expression changes (9, 10). The glutathione-S-transferase activity status was also modulated by the supplement displaying antioxidant effects. The preclinical studies also display enhanced metabolism promoted by molecules such as PPARα, adenosine monophosphate-activated protein kinase (AMPK)-PGC-1α, and Mitochondrial transcription factor A (TFAM) signaling pathways. Notably, the cortisol/C-reactive protein ratio, a well-characterized marker of the hypothalamic–pituitary–adrenal axis immune interface status, was found to be modulated by the supplement (9, 10).

Based on these preclinical studies and aiming to build up the evidence pyramid in this rapidly growing area of research, we have initiated a double-blind randomized clinical trial applying this supplement formula to participants who were overweight or obese. In this clinical trial, we began by focusing on evaluating clinical, biochemical, and anthropometric parameters. This is relevant because it brings scientific criteria for the investigation and analysis of parameters affordable to clinical practitioners who work in the area of obesity prevention and treatment. In this way, we hope to generate knowledge and scientific support for the advancement and updating of clinical practice in this research area.

Regarding the results of our randomized double-blind clinical trial, it is important to note that there was no indication of dietary interventions, encouragement of physical activity, or sort of a change in the participants’ lifestyle. Therefore, our results reflect the effects that can be directly and exclusively attributed to the consumption of the nutraceutical formula applied in the study.

The characterization of the anthropometric and demographic data shows that there are no significant differences between the groups at the beginning of the study. This is relevant to state the similarity of both groups in our sample to guarantee the reliability of data. Analyzing the anthropometrics among the supplementation groups at different time points, it is noteworthy that the WC reduction in both groups was promoted by the supplementation throughout the time. Despite the lack of weight loss, the reduction of WC represents a marker of decreased cardiovascular risk for the participants. It is well established that high WC reflects abdominal fat deposition, and it is strongly associated with the development of non-communicable chronic diseases (42). The indicators of abdominal obesity are better to discriminate inflammatory status and metabolic risk than the indicator and generalized obesity as BMI. In this sense, considering the cutoff points of WHtR and WHR preconized by health guidelines, it is possible to note that both markers decreased from the T0 time point to T180 time point, returning to low-risk values. The WHtR and WHR are easily collected anthropometric measures and reliable indicators for the diagnosis of obesity mainly related to increased visceral adiposity. Thus, they also represent a relevant clinical parameter to assess cardiovascular risk that is strongly related to visceral fat deposition. When increased, WHtR is a good predictor of metabolic syndrome and appears to be directly proportional to the inflammatory indicators and directly correlated with poor eating habits (12–14). Also, the neck circumference was slightly reduced by both supplements at the end of the treatment compared to the beginning; this finding is related to the natural course of obesity, since the volunteers were instructed not to change their lifestyle throughout the supplementation period, in addition to not having nutritional intervention and lifestyle changes in this study. Results from previous studies show that increased neck circumference in obese adults is related to increased cardiovascular and inflammatory risks, acting as an anthropometric predictor of cardiovascular and inflammatory risks for this population (11).

In our data, the supplements with a combination of minerals, fibers, and bioactive compounds were able to restore low cardiovascular risk levels without the need for medicines or dietary interventions. It might be related to high metabolic rates and an increase in energy expenditure promoted by the supplement compounds that are described to enhance molecules related to fatty acid metabolism activation (9). In a review, (43) described the effect of reducing adiposity by intake of bioactive natural compounds in obese populations. In particular, compounds such as FOSs (44) and GOSs (45) have shown an adiposity-reducing effect through activation of β-oxidation markers, such as acetyl CoA-carboxylase (ACC) inactivation, AMPK activation, and mitochondrial oxidation *via* allosteric regulation of carnitine palmitoyl transferase 1 (CPT-1) (43). In this sense, studies that used acute bioactive compound supplementation found similar results without changes in body weight, but in other specific biomarkers (46, 47). These changes may not yet be evident in anthropometric data; however, these results allow us to believe that long-term consumption could exert more pronounced effects on weight loss or parameters such as WHtR and BMI. Thus, in clinical practice, these anthropometric changes might be a good sign of improvement in cardiometabolic status.

Considering the biochemical parameters analyzed in serum samples collected at the end of the experimental protocol, there were no significant changes in serum concentrations of total cholesterol and its fractions or TGs. The improvement of the serum lipoprotein profile through the consumption of nutraceutical compounds such as fibers and antioxidants is recognized in the literature as a strategy to control dyslipidemia and prevent the onset of cardiovascular diseases, among other metabolic changes (48). Preclinical models of obesity that chronically consumed different bioactive compounds showed improvement in serum lipids (TG, HDL-cholesterol, and LDL-cholesterol) and insulin sensitivity associated with weight (49–52). Specifically, the supplement formula with silymarin can improve insulin sensitivity and reduce pro-inflammatory cytokines such as tumor necrosis factor-alpha (TNF-α) and IL-6 in mice (9, 10). It might be attributed to silymarin compounds such as silibinin, which has been proven to be an immune modulator *in vivo*, inhibiting hepatic activation of Nuclear Factor-kappaB and consequently modulating the production of pro-inflammatory cytokines such as TNF-α, interferon-gamma, IL-4, IL-2, and Inducible nitric oxide synthase (iNOS) and upregulating anti-inflammatory cytokines such as IL-10 (35). However, these effects may also be related to the increased intake of daily fiber from FOSs and GOSs and antioxidant compounds by the supplement tested. Furthermore, these findings were not confirmed in the clinical study, showing the limitation of preclinical studies that are not able to mimic factors ranging from preexisting diseases and lifestyle, genetic predispositions and variability, and even factors such as the composition of the intestinal microbiota that can influence the response to supplement consumption.

To investigate other markers related to NAFLD, serum concentrations of AST and ALT as well as the AAR were evaluated. AST, ALT, and gamma-GT are accessible clinical markers to check for liver damage. It is known that ALT is found mainly in the hepatocyte cytoplasm, while AST is present in the mitochondria. Therefore, in cases of mild liver damage, ALT is altered in the serum, while in severe liver damage, there is an increase in AST in the bloodstream (53). Despite the absence of statistical differences in gamma-GT, it might represent an indicator of risk for advanced fibrosis in NAFLD and must be carefully watched by clinical practitioners (40). Based on this, our results allow us to suggest that there was no severe liver damage despite the mild liver damage displayed by both groups at all time points. It is important to consider that obesity and overweight are a strong risk factor for NAFLD *per se*, as the participants started the study with a high level of ALT, which was maintained until the end of the study. It is also noteworthy that the new supplement had its consumption safety reinforced, since, over time, there were no worsening or severe liver damage signs in this study. In fact, the AAR was reduced by the supplementation over time. These data are interesting, since, in alcoholic liver disease, the AAR is usually higher than 2, while in NAFLD, this index tends to be between 1 and 2. Recently, some studies have proposed the use of the AAR as the main laboratory parameter for the diagnosis of NAFLD. NAFLD is a chronic inflammatory liver disease with the evolutionary potential to cirrhosis. Currently, cases of cirrhosis in obese patients, previously diagnosed as cryptogenic, have been attributed to the evolution of NAFLD (41). Thus, AAR is an extremely relevant clinical parameter that reflects and helps in the diagnosis of hepatic complications. The supplement recovers the AAR, promoting hepatoprotective effects. This effect might be promoted by the supplement and can be explained by its rich composition. In this sense, the application of silymarin extract stands out by its hepatoprotective effects, enhancement of insulin receptor (IR) sensitivity, and immune modulation, besides its beneficial effects against cardiovascular and neurological diseases and some types of cancer among other positive effects (34). Also, the yeast β-glucan is an immunomodulatory molecule and might also contribute to the hepatoprotective effect (54). Notably, there are many drug therapies available in the pipeline that have been shown to be effective in the treatment of NAFLD/Nonalcoholic steatohepatitis (NASH) (55). However, nutraceutical supplements and integrative therapies should be considered as options for clinical frontline professionals as complementary tools and not substitutes for well-established drug treatments.

In obesity, the classic chronic low-grade inflammatory state, also known as metainflammation, is associated with increased inflammatory processes; the reactive oxygen species (ROS) is also increased. We did not find alterations in the C-reactive protein; however, this can be considered a nonspecific inflammatory marker and therefore not very sensitive to the discreet changes promoted by the metainflammation of obesity. Hyperglycemia and hyperinsulinemia are also known to trigger an inflammatory process and oxidative stress environment (56). In parallel, insulin resistance is also established by visceral fat deposition that increases the activation of the lipolysis process (57, 58). In turn, an increased flow of portal vein free fatty acids (FFAs) occurs, exposing the liver to a greater influx of FFAs modifying hepatic metabolic patterns, contributing to the maintenance of systemic hyperinsulinemia and insulin resistance (59). As observed in our results, insulin resistance comes alongside overweight/obesity and represents a persistent metabolic issue with a major concern for practitioners due to its complex management.

Accessing the endocrine profile, we found a significant reduction in cortisol levels promoted by the new supplement tested. Cortisol is a hormone well known for its key role in responding to stressful stimuli. Despite its physiological role, in obesity, it is known that high levels of cortisol promote body weight gain and increase appetite with a preference for energy-dense food, contributing to a high cardiometabolic risk. Also, chronic exposure to glucocorticoids such as cortisol is known to promote the specifically abdominal obesity metabolic syndrome and eventually cardiovascular diseases. In addition, the reduction in cortisol is related to the improvement in sleep quality, which is also directly linked to the prevalence of obesity (60). Our results demonstrate an improvement in cortisol levels, contributing to a reduction in abdominal obesity and a better metabolic prognosis without any lifestyle change intervention. In this way, the new supplement proves to be a tool capable of modulating endocrine pathways of cortisol production.

In this way, our primary clinical data allow us to conclude that the new supplement has an innovative combination of nutrients and bioactive compounds capable of promoting the improvement of biochemical and endocrine parameters in overweight/obesity. Its effect was pronounced, since it was able to promote metabolic improvement without the association of lifestyle change therapies such as diet intervention and physical activity. Although the nutrients of this supplement are accessible through a healthy diet, epidemiological data show that the consumption of these nutrients is usually deficient in a large part of the population. Thus, supplementation proves to be an important alternative to maintain adequate intake levels when the diet does not meet this demand. Therefore, the supplementation of beneficial compounds can be a valuable tool for preventing the onset of chronic diseases in the long term, avoiding the need for medication treatments. Nevertheless, these data represent an initial study of this new supplement. For more robust conclusions to be obtained, further studies are necessary with a larger sample size of both genders equally distributed. Further research also needs to address refined and in-depth biomolecular analyses that would provide a basis for a discussion on the reported clinical findings of robust cellular pathways, which might be considered another limitation of the present study. Nonetheless, the present results allow us to suggest that the parameters analyzed in this study can also be evaluated in clinical practice as an indication of endocrine and metabolic changes, bringing the research for the development of new nutraceuticals closer to the clinical practice of medical professionals, which has proven to be a major barrier between scientific research and practice application.

## Data availability statement

The raw data supporting the conclusions of this article will be made available by the authors, without undue reservation.

## Ethics statement

The studies involving human participants were reviewed and approved by the HC-FMUSP Research Ethics Committee under the CAAE number 39984320.5.0000.0068 and registered as a Clinical Trial under identification number NCT04810572 (ClinicalTrials.gov). The patients/participants provided their written informed consent to participate in this study.

## Author contributions

Conceptualization, AS and AP; Methodology, JF, DM and AP; Methodology, JF and DM; Investigation, VN and AP; Resources, VN; Data curation, ET, FP-B, and AP; Writing—original draft preparation, AS, JF and AP; writing—review, editing and visualization, AE-G, ES, and DO; Supervision, AP and JO; Project administration, AP; funding acquisition, VN. All authors contributed to the article and approved the submitted version.
